# Occurrence and Antimicrobial Susceptibility Pattern of Clinical *Escherichia coli* Isolates from Dogs in Grenada, West Indies

**DOI:** 10.3390/antibiotics15050522

**Published:** 2026-05-21

**Authors:** Erika Brewer, Kaitlin Law, Bhumika Sharma, Andy Alhassan, Erica Hazel-Ann Brathwaite, Wayne Sylvester, Kamashi Kumar

**Affiliations:** 1 School of Veterinary Medicine, St. George’s University (SGU), St. George’s, Grenada; 2 Microbiology Department, Lake Erie College of Osteopathic Medicine, Erie, PA 16509, USA; bsharma@lecom.edu; 3 Pathobiology Department, School of Veterinary Medicine, St. George’s University (SGU), St. George’s, Grenada; aalhass1@sgu.edu (A.A.); ebrathwaite@sgu.edu (E.H.-A.B.); 4 Small Animal Clinic, School of Veterinary Medicine, St. George’s University (SGU), St. George’s, Grenada; wsylvester@sgu.edu; 5 Anatomy Physiology & Pharmacology Department, School of Veterinary Medicine, St. George’s University (SGU), St. George’s, Grenada

**Keywords:** antimicrobial resistance, *Escherichia coli*, dogs, β-lactam, tetracycline, Grenada, antibacterial sensitivity test, polymerase chain reaction, next generation sequencing

## Abstract

**Background/objectives**: Infections caused by multidrug-resistant (MDR) bacteria are becoming increasingly difficult to treat with recommended antimicrobials. Considering the critical and growing challenge of antimicrobial resistance (AMR), this study aims to evaluate the antimicrobial susceptibility patterns of *Escherichia coli* clinical isolates from dogs in Grenada. This research project consists of two distinct studies: a retrospective analysis of AMR in canine *E. coli* isolates collected between 2010 and 2020, and a cross-sectional study characterizing the genotypic AMR profiles of *E. coli* isolates obtained between April and June 2023. **Methods**: A retrospective analysis of antibacterial sensitivity test (ABST) reports from canine clinical samples submitted to the Small Animal Clinic at St. George’s University (SGU), St. George’s, Grenada, between 2010 and 2020 revealed a notable prevalence of AMR among canine *E. coli* isolates. To further investigate the underlying mechanisms of this resistance, the study analyzed canine *E. coli* isolates that exhibited phenotypic resistance in ABST assays. These isolates were subsequently screened for AMR-associated genes using polymerase chain reaction (PCR) and next-generation sequencing (NGS). **Results**: The retrospective study identified 153 canine clinical isolates positive for *E. coli*. The antimicrobial drugs, imipenem, cefotaxime and ciprofloxacin were found to be highly effective against these isolates. However, a gradual increase in AMR was observed for amoxicillin–clavulanic acid (34.88%), ampicillin–sulbactam (17.31%), cephalexin (43.08%), cefpodoxime (22.31%), cephalothin (68.42%), and doxycycline (37.04%). In the prospective study, PCR analysis of resistant canine *E. coli* isolates detected the *tetA* (577 bp) and *bla*TEM (686 bp) genes. These AMR determinants were further confirmed through analysis of NGS reads and assembled contigs. Additionally, NGS-based predictions identified genes associated with resistance to aminoglycosides and potentiated sulfonamides. **Conclusions**: This study demonstrates that *E. coli* from dogs in Grenada exhibits resistance to tetracycline and several β-lactam antimicrobials. These findings underscore the need for rational antimicrobial stewardship and continuous AMR surveillance in small animal practice within the region.

## 1. Introduction

Antimicrobial resistance (AMR) is a growing global public health threat affecting both human and animal populations and is associated with increasing morbidity and mortality [1,2]. This challenge is intensified by the rising prevalence of multidrug-resistant (MDR) microorganisms worldwide [3]. The Global Antimicrobial Resistance and Use Surveillance System (GLASS) 2022 report stated that 42% of *E. coli* isolates were found to be resistant to third-generation cephalosporins [4]. Resistant *E. coli* and other microorganisms lead to extended hospital stays, increased mortality rates, and expensive treatments due to the need for more advanced drugs for patient treatment [5]. A study conducted in the United States reported that 52% of *Escherichia coli* isolates from companion animals across six regions were multidrug-resistant [6]. Moreover, resistant *E. coli* poses a significant risk of zoonotic transmission between pets and their owners, as well as dissemination into the environment.

*Escherichia coli* is a Gram-negative, facultative anaerobic, rod-shaped coliform bacterium of the genus *Escherichia*. It resides as a normal commensal member of the intestinal microbiota but, under certain physiological conditions, can shift into an opportunistic pathogenic state [7]. Because of its wide host range and increasing resistance to multiple classes of antimicrobials, *E. coli* has been designated by the World Health Organization (WHO) as one of the bacterial species posing the greatest threat to human health [8]. Commensal *E. coli* within the gut microbiota may serve as a reservoir for resistance genes because it is continuously exposed to diverse antimicrobials prescribed throughout a patient’s lifetime, allowing resistant strains to persist and transfer genetic material to pathogenic bacteria [9]. Intestinal pathogenic *E. coli* is one of the most common causes of diarrhea in canine patients. However, extra-intestinal infections caused by *E. coli* include urinary tract infections (UTIs), pyelonephritis, pyometra, mastitis, prostatitis, and septicemia [10]. *E. coli* is the most common cause of UTIs, as reported in many parts of the world [11]. The updated guidelines for treating uncomplicated UTIs from the International Society for Companion Animal Infectious Diseases state that amoxicillin, or amoxicillin with clavulanic acid, is the preferred method of treatment [12]. In dogs and cats in the United States, the resistance rate to amoxicillin–clavulanic acid has been reported at 40% [6], leading to treatment failures associated with resistant *E. coli* strains.

Evolution among *E. coli* species occurs through the acquisition of foreign DNA via horizontal gene transfer. Horizontal gene transfer can occur through conjugation, transduction, or transformation, involving genetic elements such as plasmids, transposons, or bacteriophages [13]. Bacteria undergo random mutations at a rate of around 10^−6^ to 10^−9^ per nucleotide, per generation. *E. coli* is known to transfer resistance through plasmids via horizontal gene transfer [10,14]. Genetic material can encode diverse mechanisms of antimicrobial resistance. *E. coli* exhibits resistance to several classes of antimicrobials through a variety of mechanisms [5]. *E. coli* is resistant to β-lactam antimicrobials via β-lactamases, which hydrolyze their lactam ring. Class A extended-spectrum β-lactamases (ESBLs) confer resistance to extended-spectrum cephalosporins, including ceftriaxone. These enzymes hydrolyze oxyimino-cephalosporins and are derived from the β-lactamases TEM-1, TEM-2, and SHV-1; however, they remain susceptible to inhibition by clavulanic acid [15]. *E. coli* can exhibit tetracycline resistance through the *tetA* gene, a transposon-encoded gene that mediates resistance via a single-component efflux pump [16].

In a comparative study, 31% of dog–owner pairs shared multidrug-resistant *Escherichia coli* strains, indicating that resistant *E. coli* can be readily transmitted between populations [17]. A study conducted in Canada reported that 92% of 101 sampled veterinary hospitals contained environmental *E. coli*. Both non-resistant strains and *bla*CMY-2-positive *E. coli* were recovered from hospital environments [18]. These findings emphasize the need for stricter biosecurity measures to prevent hospitalized patients from acquiring *E. coli* or other infections. The widespread presence of resistant *E. coli* further highlights its significance as a One Health challenge, emphasizing the interconnected risks to human, animal, and environmental health [19].

Antimicrobial therapy exerts significant selective pressure on *E. coli*, leading to the development of AMR. Routine and inappropriate antimicrobial use further contribute to the spread of resistance [20]. Consequently, commensal gut *E. coli* may acquire multidrug resistance over a patient’s lifetime, influenced by the frequency and type of antimicrobials administered in clinical practice [9].

Despite this growing concern, data on AMR patterns in bacterial isolates from Grenada remain scarce, limiting the ability to guide evidence-based antimicrobial use. Hence, the present study aimed to investigate antibacterial susceptibility patterns and identify the genes that confer AMR in canine *E. coli* isolates from Grenada.

## 2. Results

The results of this study originate from a retrospective and a prospective investigation: (i) a retrospective analysis of AMR in canine *E. coli* isolates collected between 2010 and 2020, and (ii) a prospective study involving *E. coli* isolates collected between April and June 2023 for genotypic AMR analysis. The outcomes of these studies are detailed in this section.

### 2.1. Retrospective Analysis of Antimicrobial Susceptibility Pattern of E. coli

**Figure 1:**
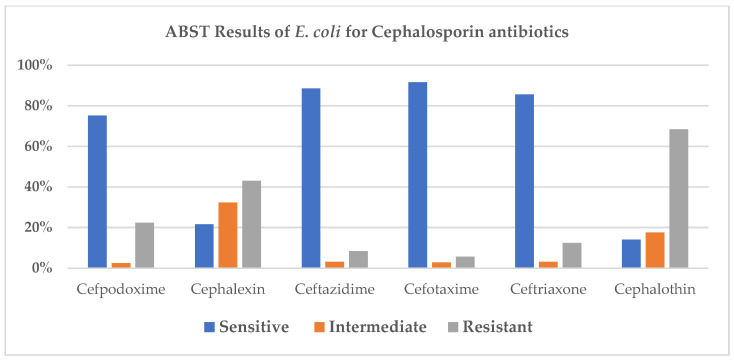
Antibacterial sensitivity test (ABST) results of *E. coli* for Cephalosporin antibiotics.

**Figure 2:**
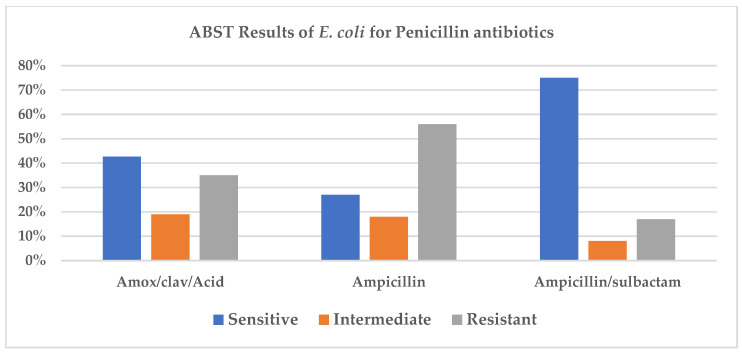
Antibacterial sensitivity test (ABST) results of *E. coli* for β-lactam antibiotics.

**Figure 3:**
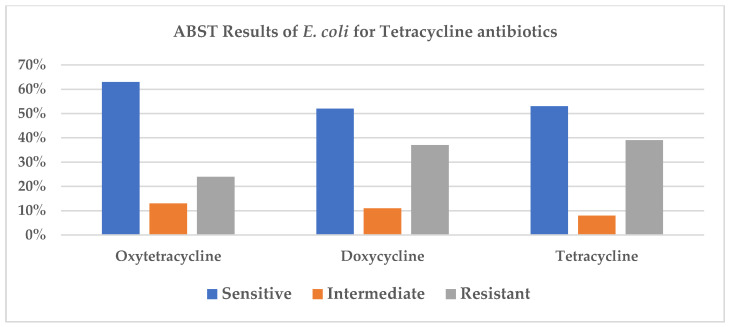
Antibacterial sensitivity test (ABST) results of *E. coli* for tetracycline antibiotics.

Antibacterial susceptibility test (ABST) reports for clinical samples collected from canine patients between 2010 and 2020 were retrieved from the AVImark patient record system for retrospective analysis. Over the study period, 153 *Escherichia coli*–positive isolates were identified. Most positive samples were from urinary tract infections. However, pyometra, pyoderma, dermatitis, various wound infections, vaginitis, otitis, colitis, and septicemia were also identified in clinical cases. ABST results for the recommended antimicrobial classes for the treatment of *E. coli* infections are depicted in Figure 1 (cephalosporin), Figure 2 (β-lactam antimicrobials), and Figure 3 (tetracycline).

The cephalosporin antimicrobials demonstrating higher levels of resistance were cephalothin (68.42%) and cephalexin (43.08%) (Figure 1). The commonly used antimicrobial amoxicillin + clavulanic acid displayed a higher resistance rate of 34.88%, with intermediate resistance being 19.38% (Figure 2).

Tetracycline class antibiotics—doxycycline, oxytetracycline, and tetracycline—showed variable sensitivity patterns against *E. coli* isolates. Descriptive data indicate year-to-year variation in resistance levels (Figure 3).

#### Antimicrobials with the Maximal Antibacterial Efficacy Against *E. coli*

**Figure 4:**
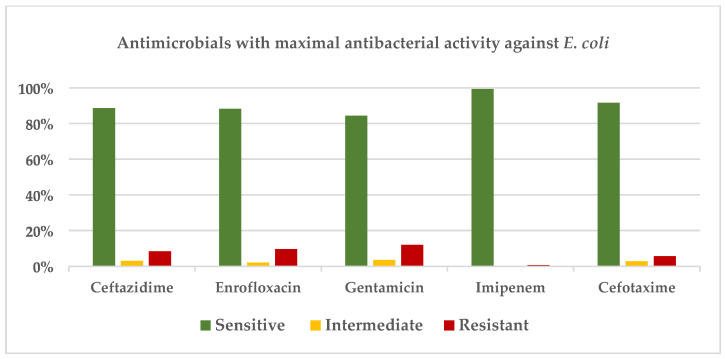
Antimicrobials with the maximal antibacterial efficacy against *E. coli*.

The antimicrobial with the lowest resistance was imipenem (0.07%). Other antimicrobials with high sensitivity included cefotaxime (91.55%), ceftazidime (88.55%), ceftriaxone (85.57%), enrofloxacin (88.28%), and gentamicin (84.40%). The results are presented in Figure 4.

Resistance to commonly used β-lactam and tetracycline antimicrobials generally increased over the study period, demonstrating variable sensitivity patterns. Of the 153 isolates, 59 met the criteria for multidrug resistance (MDR), with resistance to three or more classes of antimicrobials, and the finding that 39% of infections were classified as MDR remains a significant concern.

### 2.2. Phenotypic Analysis Outcomes

Based on the ABST results, 18 canine *E. coli* isolates that were resistant to β-lactam antibiotics and tetracycline were selected for genotypic analysis of antimicrobial resistance.

### 2.3. Genotypic AnalysisOutcomes

#### 2.3.1. Polymerase Chain Reaction (PCR)

**Figure 5:**
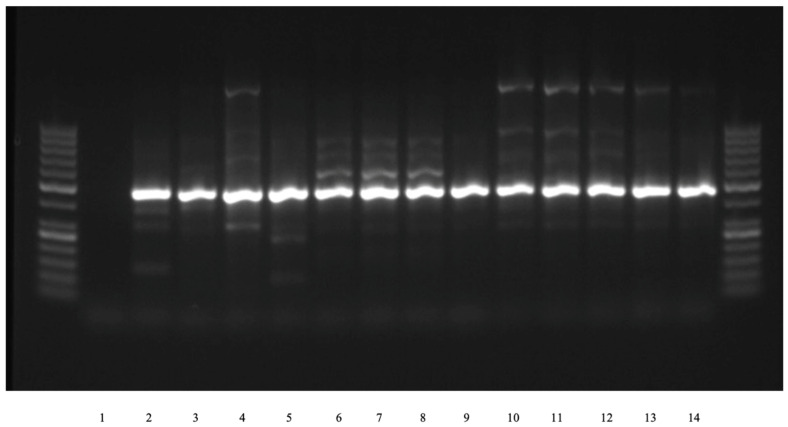
PCR-Based Identification of *E. coli* Isolates. Lane 1: negative control; lane 2: positive control plasmid for ATCC 25922 *E. coli*; lanes 3–14: unknown *E. coli* DNA samples. Lanes 3–14 have a band present at the corresponding location, same as the positive control, ATCC 25922 *E. coli*.

The suspected *E. coli* isolates were subjected to PCR analysis using universal primer pair 16s2F and 16s4R [21] that amplified approximately 500 bp gene fragment and further confirmed via Sanger sequencing. ATCC 25922 *E. coli* was used as a positive control, and molecular-grade water as a negative control for PCR amplification. The PCR and sequencing results determined that 15/18 resistant isolates (83.3%) were pure canine *E. coli* isolates (Figure 5).

**Figure 6:**
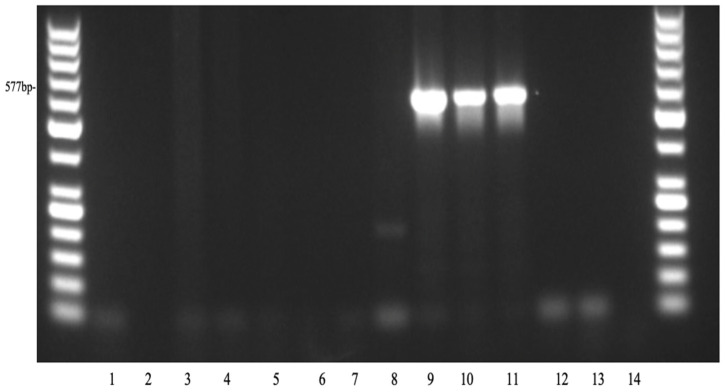
Detection of the *tetA* gene in *E. coli* isolates with conventional PCR. Lanes 1–3: Negative and positive controls; lanes 4–14: canine *E. coli* DNA samples, respectively; lanes 9–11: show a band corresponding to the 577 bp marker for *tetA*.

**Figure 7:**
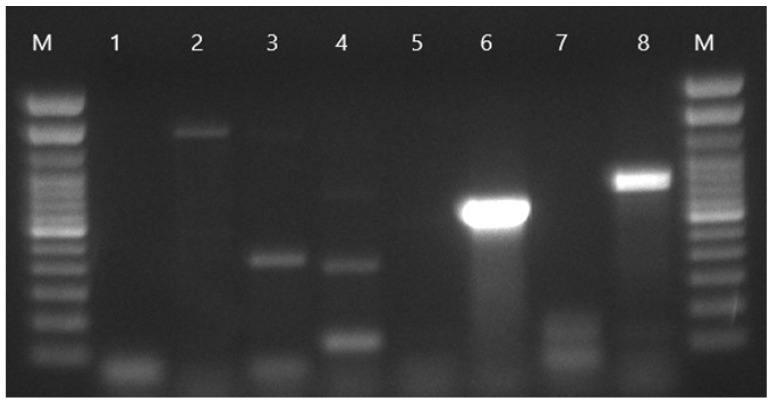
Detection of the *bla*TEM gene in *E. coli* isolates with conventional PCR. Lane 1: negative control; lane 2: positive control plasmid for ATCC 25922 *E. coli*; lanes 3–8: unknown *E. coli* DNA samples. Lane 6: showing a band corresponding to the 686 bp marker for *bla*TEM.

In PCR amplification, the *tetA* gene is typically detected as a product of approximately 577 bp, which serves as a molecular marker for its presence (Figure 6) and the *bla*TEM gene appears as a band of approximately 686 bp (Figure 7).

#### 2.3.2. Next Generation Sequencing (NGS) Results

##### Genome Annotation

The *Escherichia coli* ARX736_pass_finalAll_Flye genome was annotated using the RAST tool kit (RASTtk) and assigned the unique genome identifier 562.139494. This genome belongs to the superkingdom Bacteria and was annotated using genetic code 11. Its taxonomic classification is as follows:

cellular organisms > Bacteria > Pseudomonadota > Gammaproteobacteria > Enterobacterales > Enterobacteriaceae > *Escherichia* > *Escherichia coli*

**Figure 8:**
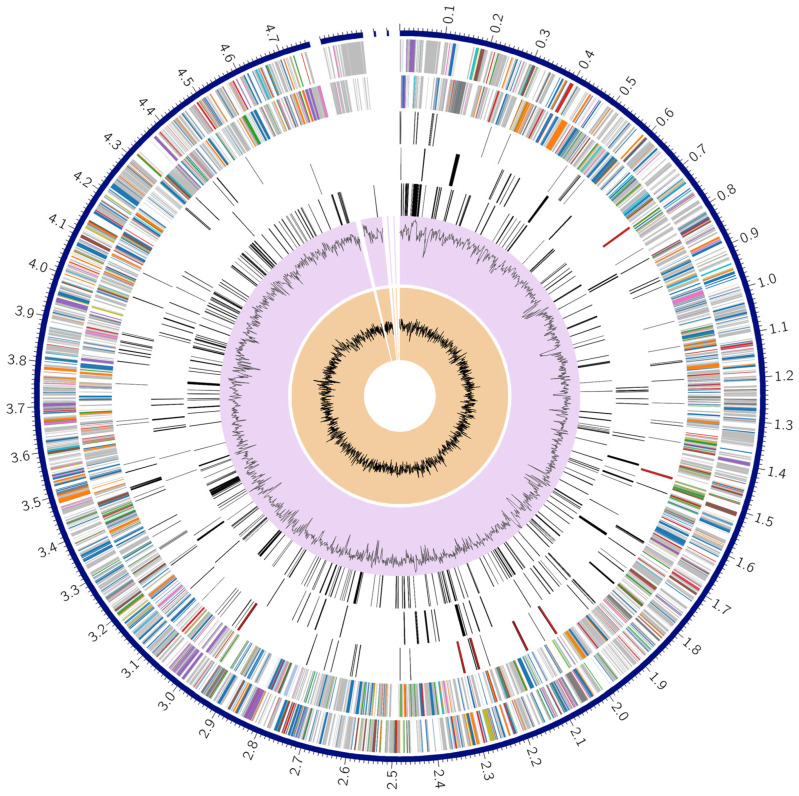
Distribution of the genome annotations of *E. coli*. The colors of the CDS on the forward and reverse strand indicate the subsystem that these genes belong to. The distribution is displayed in the pie chart below (Figure 9).

A circular graphical display of the distribution of the genome annotations is provided (Figure 8). This includes, from outer to inner rings, the contigs, CDS on the forward strand, CDS on the reverse strand, RNA genes, CDS with homology to known antimicrobial resistance genes, CDS with homology to known virulence factors, GC content, and GC skew.

**Figure 9:**
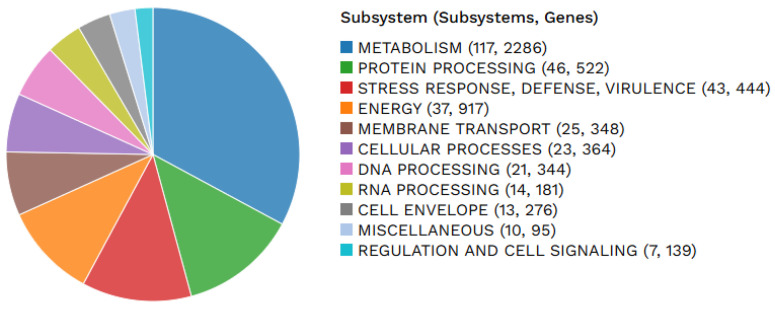
Pie chart detailing the distribution of genes across various functional subsystems within a specific organism’s genome.

##### Genes Contributing to *E. coli* Antimicrobial Resistance

The presence of the AMR-conferring genes *tetA* and *bla*TEM was confirmed through analysis of NGS reads and assembled contigs from the *E. coli* isolates. Additionally, NGS data predicted the presence of genes associated with resistance to aminoglycosides, sulfonamides, and trimethoprim (Table 1).

The Genome Annotation Service in PATRIC uses a k-mer-based method for AMR gene detection, which utilizes PATRIC’s curated collection of representative AMR gene sequence variants. This approach assigns each AMR gene a functional annotation, the broad mechanism of antibiotic resistance, the respective drug class, and, when possible, the specific antibiotic to which it confers resistance. However, the presence of AMR-related genes, even full-length genes in a given genome, does not necessarily indicate a resistant phenotype. It is essential to consider the specific resistance mechanisms involved, particularly the presence or absence of SNPs that confer resistance. A summary of the AMR genes annotated in this genome and their corresponding resistance mechanisms is provided in Table 2.

## 3. Discussion

Antimicrobial resistance is a critical global health challenge affecting many countries worldwide. However, information on AMR in Grenada remains limited. Therefore, understanding the AMR patterns among bacterial isolates in Grenada is essential. This study has characterized the resistance patterns of canine *E. coli* isolates in Grenada. In this investigation, the retrospective analysis provided insight into phenotypic resistance patterns, while the prospective study enabled the identification of AMR determinants among the canine *E. coli* isolates. The results provide clear evidence that antimicrobial resistance is increasing within *E. coli* populations associated with infection. Higher levels of resistance to the broad-spectrum antimicrobials ampicillin and amoxicillin were observed, aligning with previous findings [6]. Although current treatment guidelines recommend amoxicillin or amoxicillin–clavulanic acid for uncomplicated UTIs [12], the patterns identified in this study suggest that these recommendations may need to be reassessed to ensure optimal animal care in Grenada.

Cephalosporin antibiotics, including cephalexin and cephalothin, showed higher levels of AMR among the *E. coli* isolates. This may be due to the widespread presence of CTX–M-type extended-spectrum β-lactamases (ESBLs) in bacterial communities worldwide, particularly in *E. coli* [22]. Tetracycline also exhibited a high level of resistance among the tested antimicrobials. This could be associated with the drug’s broad-spectrum activity and frequent use, which has led to the emergence of tetracycline-resistant bacterial strains [23,24]. This result is consistent with earlier reports from *E. coli* studies in Grenada, where AMR to tetracycline (23.4%) and cephalothin (13.2%) was documented [25]. The antimicrobials that demonstrated the lowest levels of emerging resistance should be reserved for emergency use in *E. coli*–positive patients to slow the development of resistance to these drugs. Imipenem is not used in routine veterinary practice in Grenada; however, it was included in the antimicrobial susceptibility testing panel to characterize the susceptibility profile of the isolates. As a critically important antimicrobial, its activity should be interpreted within the framework of antimicrobial stewardship and established veterinary guidelines. Figure 4 highlights the antimicrobials that should be withheld unless there is a strong clinical justification for their use.

The phenotypically resistant *E. coli* isolates carried antimicrobial resistance (AMR) genes associated with β-lactam and tetracycline resistance. Further molecular characterization of *E. coli* is necessary to track the movement of AMR determinants between companion animals and humans and to better assess zoonotic potential. Therefore, isolates exhibiting phenotypic resistance in antimicrobial susceptibility testing (ABST) were subjected to polymerase chain reaction (PCR) and next-generation sequencing for genotypic analysis.

In this study, the *tetA* gene was identified as the primary contributor to tetracycline resistance in *Escherichia coli* isolated from canines in Grenada. Of the 15 *E. coli* isolates examined, only 3 isolates (20%) carried the *tetA* gene. This relatively low prevalence suggests that tetracycline resistance within this sample set may rely on alternative mechanisms or resistance genes beyond *tetA*. The *tetA* gene encodes a membrane-associated efflux pump that actively exports tetracycline from the bacterial cell, thereby lowering intracellular drug concentrations and enabling survival in the presence of the antibiotic. Tetracycline resistance rates in *E. coli* vary around the world, from 94.7% in a hospital in Al Muthanna [26], to an almost 100% resistance rate in Panama [27], to 75.6% in Grenada [28]. The widespread resistance of *E. coli* to tetracyclines could be attributed to several AMR genes [26]. Tetracycline resistance genes are typically located on plasmids and transposons, enabling their transfer through conjugation [29]. Among Gram-negative bacteria, the most frequently detected *tet* genes are those encoding efflux pumps, including *tetA*, *tetB*, *tetC*, *tetD*, and *tetG* [30]. Several research studies have confirmed that *tetA* is a major antimicrobial resistance gene commonly associated with tetracycline resistance in *E. coli* [27,31,32]. Our findings are consistent with previous studies on tetracycline resistance.

The AMR genes for β-lactam antimicrobial, *blaTEM* was detected in the canine isolates of *E. coli*. *blaTEM* is responsible for degrading the β-lactam ring and extended-spectrum β-lactamase action. A study reported that among 42 ampicillin-resistant *E. coli* isolates, obtained from 187 samples of raw milk and the two most popular cheeses in Egypt, 40 isolates (94.23%) harbored the *blaTEM* gene, whereas 9 isolates (21.42%), and 3 isolates (7.14%) harbored *blaCTX*-*M* and *blaSHV*, respectively [33]. Extended-spectrum β-lactamase (ESBL)-producing isolates carried both the *blaCTX*-*M* and *blaTEM* genes [34]. Pathogenic *E. coli* strains isolated from raw milk samples and unpasteurized cheese samples were detected for the β-lactamase gene *blaTEM* [35]. The most frequent genotypic antimicrobial resistance patterns were *blaCTX*-*M*–*sul1*–*tetA*–*tetB*–*blaTEM* [34]. The predominant ESBL genes identified in *E. coli* isolates resistant to cefotaxime were TEM and CTX-M [36,37]. The ESBL genes are encoded on plasmids or chromosomal DNA [36,38]. The TEM β-lactamase was the first plasmid-mediated enzyme, from which many of the ESBLs have evolved [39]. As these ESBLs are effective in hydrolyzing the third generation of cephalosporins, the rational use of antimicrobial agents is critically important. *E. coli* strains isolated from humans have consistently shown high levels of resistance to ampicillin, trimethoprim/sulfamethoxazole, and ciprofloxacin, along with marked peaks in ESBL production and multidrug resistance, highlighting the issue from a One Health perspective [40].

The conventional PCR findings in our study were validated using next-generation sequencing (NGS). This NGS-based assay focuses on selected short DNA regions and enables the simultaneous detection of multiple resistance genes within a single sample, while sequencing only a few kilobases of the genome [41]. NGS technologies enabled AMR detection through high-resolution genotyping [42,43]. NGS, in conjunction with conventional PCR assays, demonstrates a powerful approach to comprehensively identify and characterize resistance genes [42], offering a complete picture of the genetic mechanisms driving AMR in *E. coli* isolated from canine clinical samples in Grenada. Genomic analysis revealed additional resistance genes against aminoglycosides, sulfonamides, and trimethoprim, suggesting potentially multifaceted resistance profiles present in the canine population on the Caribbean island.

Multidrug-resistant strains of *E. coli* are transmitted between pets and their owners due to the close sharing of space and environment [17]. Veterinary hospitals serve as critical interfaces between humans, animals, and medical practice, creating numerous opportunities for the transmission of zoonotic diseases [18,44]. Previous studies have highlighted the potential for zoonotic and environmental dissemination of resistant *Escherichia coli*. These findings are of relevance to Grenada, where the small island population is continually influenced by the influx of tourists and international students, many of whom arrive with companion animals. Such demographic dynamics increase the risk of introducing resistant strains within the local community and environment.

This study utilized isolates obtained from a single clinical setting, which represents a limitation. However, in Grenada, the Small Animal Clinic at St. George’s University serves as the primary veterinary care facility for the island, and dogs from multiple parishes routinely present to this clinic. As a result, although the samples originate from a single location in Grenada, they represent a broadly distributed canine population. It is important to note that the present study is based on clinical samples submitted for diagnostic purposes, which may not fully represent the overall canine population, including healthy or subclinical carriers. This limitation has been acknowledged in the interpretation of our findings.

AMR development in canine *E. coli* could be a major source for the introduction of resistance determinants into *E. coli* populations that affect humans. Similarly, the detection of multidrug-resistant *E. coli* and diverse β-lactamase genes in healthy domestic cats underscores an important emerging concern for public health, veterinary clinical practice, and antimicrobial-resistance surveillance within a One Health framework [45]. Thus, the cycle of zoonotic spread between humans and their pets would allow new AMR genes to develop and to enhance the MDR among bacterial organisms. Future studies incorporating randomly selected samples, larger sample sizes, and a broader range of bacterial species will be needed to more accurately assess the prevalence of AMR in Grenada.

## 4. Materials and Methods

This work was approved by the Institutional Animal Care and Use Committee protocols (IACUC-19007-R) of St George’s University (SGU), Grenada, West Indies.

The research was conducted in two sequential phases: an initial retrospective study, followed by prospective phenotypic and genotypic analyses of AMR in canine *E. coli* isolates.

### 4.1. Retrospective Study

Clinical samples were collected from canine patients presenting to the Small Animal Clinic of St. George’s University in Grenada between 2010 and 2020. Samples submitted during this period included wounds, feces, urine, exudate, and discharges. All samples were processed by the bacteriology laboratory at SGU for culture and antimicrobial susceptibility testing. The resulting reports were archived in the AVImark patient record system and subsequently retrieved for retrospective analysis in this study. Over the study period, 153 *Escherichia coli*–positive isolates were identified. The antimicrobials used in sensitivity testing included amoxicillin/clavulanic acid, cephalexin, ceftazidime, cefpodoxime, chloramphenicol, ciprofloxacin, enrofloxacin, gentamicin, imipenem, cefotaxime, oxytetracycline, ampicillin/sulbactam, doxycycline, trimethoprim/sulfa, ceftriaxone, cephalothin, ampicillin, and tetracycline.

Microsoft Excel (2013) was used for data entry and analysis. Samples were organized by year, and culture and sensitivity results were extracted from laboratory reports and compiled into tables. Annual totals were used to calculate the percentage susceptibility for each antimicrobial by dividing the number of sensitive, intermediate, or resistant results by the total number of *E. coli* isolates tested for that specific drug. Because ABST results were retrieved from the AVI system, not all isolates were tested against the same antimicrobial panel. Consequently, the number of samples tested varied between antimicrobials. To maintain consistency in reporting, resistance values are presented as percentages throughout the manuscript. A cumulative table was then generated to determine average susceptibility across all study years, enabling the identification of antimicrobial susceptibility trends over the study period.

### 4.2. Prospective Study

#### 4.2.1. Phenotypic Analysis

##### Sample Collection

Clinical samples were obtained from dogs presenting to the Small Animal Clinic, St. George’s University, Grenada, West Indies, from April to June 2023. Samples were processed for bacterial isolation. Clinical specimens were inoculated onto MacConkey agar (Remel, Lenexa, KS, USA) and incubated aerobically at 37 °C for 18–24 h. Lactose-fermenting colonies on MacConkey agar with morphology compatible with *Escherichia coli* were subcultured to obtain pure cultures. Gram staining revealed Gram-negative rods, and isolates were oxidase negative. The colonies were confirmed as *E. coli* using the analytical profile index strips (API20EBioM6rieux, Hazelwood, MO, USA) [25]. A total of 18 *E. coli* isolates were included in this study.

##### Antibacterial Sensitivity Test (ABST)

Confirmed *E. coli* isolates were subjected to antimicrobial susceptibility testing using the Kirby–Bauer disk diffusion method [46] on Mueller–Hinton (MH) agar, following the Clinical and Laboratory Standards Institute (CLSI) guidelines. A 0.5 McFarland bacterial suspension was prepared from 18 to 24 h cultures of each *E. coli* isolate and uniformly inoculated onto MH agar plates. Commercial antibiotic disks were applied to the agar surface, and plates were incubated aerobically at 37 °C for 18–24 h. The antimicrobial panel included representatives of the β-lactam and tetracycline classes to assess clinically relevant resistance patterns in companion-animal practice. After incubation, inhibition zone diameters were measured and interpreted to classify isolates as susceptible, intermediate, or resistant. Multidrug resistance (MDR) refers to isolates that exhibit resistance to at least one antimicrobial agent in three or more antimicrobial classes. All resistant isolates were preserved for subsequent molecular characterization.

#### 4.2.2. Genotypic Analysis

##### Conventional Polymerase Chain Reaction (PCR)

The phenotypically multidrug-resistant *E. coli* isolates were subjected to PCR and sequencing to confirm their species identity. This molecular verification step ensured that all isolates included in the analysis were accurately classified as *E. coli*.

Deoxyribonucleic Acid (DNA) Extraction from the Bacterial Isolates:

DNA was extracted from the bacterial isolates using the DNeasy Blood & Tissue Kit (Qiagen, Hilden, Germany). The extraction protocols provided by the kit manufacturers were followed. American Type Culture Collection (ATCC for *Escherichia coli* (ATCC 25922) and ATCC for *Klebsiella pneumoniae* (ATCC 700603) were used as positive controls. ATCC 700603 was used as a positive control as it contains AMR genes for β-lactam resistance. Genomic DNA from these reference strains was extracted separately using the DNeasy Blood & Tissue Kit (Qiagen, Hilden, Germany). The purity and concentrations of the gDNA were detected using the NanoDrop™ 2000 Spectrophotometer (Eppendorf, Hauppauge, NY, USA). The extracted DNA samples were then stored at −20 °C until further molecular and genetic characterizations were performed.

Phenotypically resistant *E. coli* isolates were subjected to conventional PCR to determine the presence of tetracycline resistance genes (*tetA*, *tetB*) and β-lactamase genes (*bla*TEM, *bla*SHV, *bla*CTX-*M*) in their extracted genomic DNA (Table 3).

**PCR protocol**:

**16s rRNA (*E. coli*)**: The PCR preparation and cycling conditions were as follows: A total of 25 µL reaction mixture was prepared containing a final concentration of 1× PCR reaction buffer, 200 µM of dNTPs, 1.5 mM of MgCl_2_, 0.5 μM of the forward and reverse primers, 1.25 U of Taq DNA polymerase, and 1 μL (~10 ng to 30 ng) of DNA template. Samples were initially heated for 5 min at 94 °C, followed by 30 amplification cycles of 1 min at 94 °C (denaturation), 1 min at 60 °C (primer annealing), and 1 min at 72 °C (primer extension). A final elongation step (72 °C for 7 min) followed the final amplification cycle [21].

***tetA* and *tetB***: The PCR preparation and cycling conditions for both assays, which were performed separately were as follows: A total of 25 µL reaction mixture was prepared containing a final concentration of 1× GoTaq^®^ G2 Green Master Mix (Promega Corporation, Madison, WI, USA) (containing 400 µM of each dNTPs, and 3 mM of MgCl_2_), 0.5 µM of the forward and reverse primers, and 2 μL of DNA template. Samples were initially heated for 4 min at 95 °C, followed by 40 amplification cycles of 1 min at 95 °C (denaturation), 1 min at 56 °C (primer annealing), and 1 min at 72 °C (primer extension). A final elongation step (72 °C for 7 min) followed the final amplification cycle [48].

***bla*_TEM_, *bla*_SHV_, *and bla*_CTX-M_**: The PCR sample preparation protocol for these three genes was similar and was as follows: A total of 25 µL reaction mixture was prepared containing a final concentration of 1× GoTaq^®^ G2 Green Master Mix (containing 400 µM of each dNTP and 3 mM of MgCl_2_), 0.4 µM of the forward and the reverse primers, and 2 μL of DNA template. The PCR cycling conditions differed in the primer annealing step as follows: samples were initially heated for 4 min at 95 °C, followed by 40 amplification cycles of 1 min at 95 °C (denaturation), primer annealing for 1 min at 58 °C (*bla*TEM), 58.5 °C (*bla*CTX-M), 55 °C (*bla*SHV) and 1 min at 72 °C (primer extension). A final elongation step (72 °C for 7 min) followed the final amplification cycle [50].

##### Next Generation Sequencing (NGS)

The genomic DNA extract from the resistant *E. coli* isolate, which showed specific amplification of PCR fragments within the AMR genes tetA and *bla*TEM, was sequenced on the Oxford Nanopore Technologies MinION™ platform (MinKNOW software). The web-server-based tool ResFinder (v4.6.0) was used to identify the presence of acquired antimicrobial resistance genes from the raw base called Nanopore reads generated from the sequencing run. Reads were de novo assembled and polished using the NGS assemblers Flye (v2.9.1) and Racon (v1.4.20). The resulting whole-genome assembly was scanned against the ResFinder, PlasmidFinder, and PointFinder databases to search for AMR genes using the staramr (0.10.0) tool.

## 5. Conclusions

This study documented that *E. coli* resistance rates in Grenada were higher than expected, particularly within antimicrobial classes routinely used to treat infections. This upward trend highlights the growing challenge of managing *E. coli* infections in dogs using commonly prescribed antimicrobials. The findings provide current antimicrobial resistance (AMR) patterns of *E. coli* isolates in Grenada and contribute to the broader global discourse on antimicrobial stewardship. Importantly, antimicrobials that remain effective should be reserved as last-line options to mitigate the risk of multidrug-resistant pathogen emergence.

The study’s findings highlight the importance of continued surveillance of AMR in veterinary pathogens, as the transfer of resistant bacteria between animals and humans poses a significant public health risk. Multidrug resistance and the resulting AMR represent a One Health challenge. Therefore, responsible therapeutic decision-making regarding antimicrobial use remains a critical component of effective disease management.

## Figures and Tables

**Table 1:** AMR prediction by NGS data analysis.

Antibiotic	Resistance Gene	Identity (%)		Alignment Length/Gene Length	Coverage	Phenotype
		ResFinder on Reads	Staramr on Contigs			
Tetracycline	*tetA*	100.00	99.68	1200/1200	100.00	Doxycycline, Tetracycline
β-lactam	*bla*TEM-1B	99.77	99.42	861/861	100.00	Amoxicillin, Ampicillin, Cephalothin, Piperacillin, Ticarcillin
Aminoglycoside	aph(6)-Id	100.00	99.28	837/837	100.00	Streptomycin
	aph(3″)-Ib	99.88	99.5	804/804	100.00	Streptomycin
Sulphonamide	sul2	100.00	99.39	816/816	100.00	Sulfamethoxazole
Trimethoprim	dfrA8	100.00	99.8	510/510	100.00	Trimethoprim

**Table 2:** Antimicrobial resistant genes and their respective AMR mechanisms.

AMR Mechanism	Genes
Antibiotic activation enzyme	KatG
Antibiotic inactivation enzyme	APH(3″)-I, APH(6)-Ic/APH(6)-Id, BlaEC family, TEM family
Antibiotic resistance gene cluster, cassette, or operon	MarA, MarB, MarR
Antibiotic target in susceptible species	Alr, Ddl, dxr, EF-G, EF-Tu, folA, Dfr, folP, gyrA, gyrB, inhA, fabI, Iso-tRNA, kasA, MurA, rho, rpoB, rpoC, S10p, S12p
Antibiotic target protection protein	BcrC
Efflux pump conferring antibiotic resistance	AcrAB-TolC, AcrAD-TolC, AcrEF-TolC, AcrZ, EmrAB-TolC, EmrD, EmrKY-TolC, MacA, MacB, MdfA/Cmr, MdtABC-TolC, MdtEF-TolC, MdtL, MdtM, SugE, Tet(A), TolC/OpmH
Gene conferring resistance via absence	gidB
Protein altering cell wall charge conferring antibiotic resistance	GdpD, PgsA
Regulator modulating expression of antibiotic resistance genes	AcrAB-TolC, EmrAB-TolC, GadE, H-NS, OxyR

**Table 3:** AMR resistant gene sequence primers used for conventional PCR.

Target Gene	Gene Sequence	Fragment Size (bp)	References
16s rRNA (for testing *E. coli*)	16s2F: CCTACGGRSGCAGCAG 16s4R:GGACTACCMGGGNTATCTAATCCKG	500	[21]
*tetA* [47] (tetracycline)	F: GGTTCACTCGAACGACGTCA R: CTGTCCGACAAGTTGCATGA	577	[48]
*tetB* [47] (tetracycline)	F: CCTCAGCTTCTCAACGCGTG R: GCACCTTGCTCATGACTCTT	815	[48]
*bla*TEM [49] (*β*-lactam)	F: GCTCACCCAGAAACGCTGGT R: CCATCTGGCCCCAGTGCTGC	686	[50]
*bla*SHV [49] (*β*-lactam)	F: CCCGCAGCCGCTTGAGCAAA R: CATGCTCGCCGGCGTATCCC	733	[50]
*bla*CTX-M [49] (*β*-lactam)	F: SCSATGTGCAGYACCAGTAA R: ACCAGAAYVAGCGGBGC	585	[50]

## Data Availability

All data generated or analyzed during this study are included in this published article.

## Abbreviations

The following abbreviations are used in this manuscript:

AMR	Antimicrobial Resistance
ATCC	American-Type Culture Collections
ABST	Antibacterial Sensitivity Test
*E. coli*	*Escherichia coli*
ESBL	Extended spectrum Beta Lactamases
MDR	Multi drug resistant
GLASS	Global Antimicrobial Resistance and Use Surveillance System
bp	base pairs
DNA	Deoxy ribonucleic acid
RNA	Ribonucleic caid
MH	Mueller Hinton Agar
NGS	Next generation sequencing
CDS	Coding sequence
PCR	Polymerase chain reaction
SGU	Saint George’s University
PATRIC	The PathoSystems Resource Integration Center
UTI	Urinary tract infections
WHO	World Health Organization
RAST	Rapid Annotation using subsystem Technology
GC	Guanine and cytosine
SNP	Single-Nucleotide Polymorphism
IACUC	Institutional Animal Care and Use Committee

## References

[1] Upadhyay S., Chakravarti A., Bharara T., Yadav S. (2020). CSE (Ceftriaxone + Sulbactam + Disodium Edta): A Possible Solution to the Global Antimicrobial Resistance Pandemic. J. Pure Appl. Microbiol..

[2] European Centre for Disease Prevention and Control (ECDC) (2023). Antimicrobial Resistance in the EU/EEA (EARS-Net)—Annual Epidemiological Report for 2022.

[3] Tenover F.C. (2006). Mechanisms of antimicrobial resistance in bacteria. Am. J. Med..

[4] World Health Organization (2022). Global Antimicrobial Resistance and Use Surveillance System (GLASS) Report 2022.

[5] Raboisson D., Ferchiou A., Sans P., Lhermie G., Derville M. (2020). The economics of antimicrobial resistance in veterinary medicine: Optimizing societal benefits through mesoeconomic approaches from public and private perspectives. One Health.

[6] Thungrat K., Price S.B., Carpenter D.M., Boothe D.M. (2015). Antimicrobial susceptibility patterns of clinical *Escherichia coli* isolates from dogs and cats in the United States: January 2008 through January 2013. Vet. Microbiol..

[7] Braz V.S., Melchior K., Moreira C.G. (2020). *Escherichia coli* as a Multifaceted Pathogenic and Versatile Bacterium. Front. Cell. Infect. Microbiol..

[8] Galindo-Méndez M. (2020). Antimicrobial Resistance in *Escherichia coli*. E. coli Infections—Importance of Early Diagnosis and Efficient Treatment.

[9] Szmolka A., Nagy B. (2013). Multidrug resistant commensal *Escherichia coli* in animals and its impact for public health. Front. Microbiol..

[10] Poirel L., Madec J.Y., Lupo A., Schink A.K., Kieffer N., Nordmann P., Schwarz S. (2018). Antimicrobial Resistance in *Escherichia coli*. Microbiol. Spectr..

[11] Irizarry R., Amadi V., Brathwaite-Syl-vester E., Nicholas-Thomas R., Shar-ma R., Hariharan H. (2016). Update on urinary tract infections in dogs in a troical island and antimicrobial susceptibility of *Escherichia coli* isolates for the period 2010–2016. Vet. Med. Open J..

[12] Weese J.S., Blondeau J., Boothe D., Guardabassi L.G., Gumley N., Papich M., Jessen L.R., Lappin M., Rankin S., Westropp J.L. (2019). International Society for Companion Animal Infectious Diseases (ISCAID) guidelines for the diagnosis and management of bacterial urinary tract infections in dogs and cats. Vet. J..

[13] Javadi M., Bouzari S., Oloomi M. (2017). Horizontal Gene Transfer and the Diversity of *Escherichia coli*. Escherichia coli—Recent Advances on Physiology, Pathogenesis and Biotechnological Applications.

[14] Aly S.A., Debavalya N., Suh S.-J., Oryazabal O.A., Boothe D.M. (2012). Molecular mechanisms of antimicrobial resistance in fecal *Escherichia coli* of healthy dogs after enrofloxacin or amoxicillin administration. Can. J. Microbiol..

[15] Shaheen B.W., Nayak R., Foley S.L., Kweon O., Deck J., Park M., Rafii F., Boothe D.M. (2011). Molecular characterization of resistance to extended-spectrum cephalosporins in clinical *Escherichia coli* isolates from companion animals in the United States. Antimicrob. Agents Chemother..

[16] Lee A., Mao W., Warren M.S., Mistry A., Hoshino K., Okumura R., Ishida H., Lomovskaya O. (2000). Interplay between efflux pumps may provide either additive or multiplicative effects on drug resistance. J. Bacteriol..

[17] Carvalho A.C., Barbosa A.V., Arais L.R., Ribeiro P.F., Carneiro V.C., Cerqueira A.M.F. (2016). Resistance patterns, ESBL genes, and genetic relatedness of *Escherichia coli* from dogs and owners. Braz. J. Microbiol..

[18] Murphy C.P., Reid-Smith R.J., Boerlin P., Weese J.S., Prescott J.F., Janecko N., Hassard L., McEwen S.A. (2010). *Escherichia coli* and selected veterinary and zoonotic pathogens isolated from environmental sites in companion animal veterinary hospitals in southern Ontario. Can. Vet. J..

[19] Holvoet K., Sampers I., Callens B., Dewulf J., Uyttendaele M. (2013). Moderate prevalence of antimicrobial resistance in *Escherichia coli* isolates from lettuce, irrigation water, and soil. Appl. Environ. Microbiol..

[20] Boothe D.M., Debavalya N. (2009). Impact of routine antimicrobial therapy on canine fecal *Escherichia coli* antimicrobial resistance: A pilot study. Intern. J. Appl. Res. Vet. Med..

[21] Cahill R., Tan S., Dougan G., O’gaora P., Pickard D., Kennea N., Sullivan M., Feldman R., Edwards A. (2005). Universal DNA primers amplify bacterial DNA from human fetal membranes and link Fusobacterium nucleatum with prolonged preterm membrane rupture. Mol. Hum. Reprod..

[22] Pitout J.D., Laupland K.B. (2008). Extended-spectrum beta-lactamase-producing Enterobacteriaceae: An emerging public-health concern. Lancet Infect. Dis..

[23] Gow S.P., Waldner C.L., Harel J., Boerlin P. (2008). Associations between antimicrobial resistance genes in fecal generic *Escherichia coli* isolates from cow-calf herds in western Canada. Appl. Environ. Microbiol..

[24] Speer B.S., Shoemaker N.B., Salyers A.A. (1992). Bacterial resistance to tetracycline: Mechanisms, transfer, and clinical significance. Bacterial resistance to tetracycline: Mechanisms, transfer, and clinical significance. Clin. Microbiol. Rev..

[25] Amadi V.A., Hariharan H., Amadi O.A., Matthew-Belmar V., Nicholas-Thomas R., Perea M.L., Carter K., Rennie E., Kalasi K., Alhassan A. (2019). Antimicrobial resistance patterns of commensal *Escherichia coli* isolated from feces of non-diarrheic dogs in Grenada, West Indies. Vet. World.

[26] Jassim E.K., Badi A., Jawad R., Al-Salihi K.A. (2019). Isolation and characterization of Oxytetracyclin resistance *E. coli* in Al Muthanna Veterinary hospital using Multiplex PCR. Mirror Res. Vet. Sci. Anim..

[27] Ramírez-Bayard I.E., Mejía F., Medina-Sánchez J.R., Cornejo-Reyes H., Castillo M., Querol-Audi J., Martínez-Torres A.O. (2023). Prevalence of Plasmid-Associated Tetracycline Resistance Genes in Multidrug-Resistant *Escherichia coli* Strains Isolated from Environmental, Animal and Human Samples in Panama. Antibiotics.

[28] Hariharan H., Brathwaite-Sylvester E., Matthew Belmar V., Sharma R. (2016). Bacterial Isolates from Urinary Tract Infection in Dogs in Grenada, and Their Antibiotic Susceptibility. Open J. Vet. Med..

[29] Guillaume G., Verbrugge D., Chasseur-Libotte M.-L., Moens W., Collard J.-M. (2000). PCR typing of tetracycline resistance determinants (Tet A-E) in Salmonella enterica serotype Hadar and in the microbial community of activated sludges from hospital and urban wastewater treatment facilities in Belgium. FEMS Microbiol. Ecol..

[30] Skocková A., Cupáková S., Karpísková R., Janstová B. (2012). Detection of tetracycline resistance genes in *Escherichia coli* from raw cow’s milk. J. Microbiol. Biotech. Food Sci..

[31] Schwaiger K., Hölzel C., Bauer J. (2010). Resistance gene patterns of tetracycline resistant *Escherichia coli* of human and porcine origin. Vet. Microbiol..

[32] Jahantigh M., Samadi K., Dizaji R.E., Salari S. (2020). Antimicrobial resistance and prevalence of tetracycline resistance genes in *Escherichia coli* isolated from lesions of colibacillosis in broiler chickens in Sistan, Iran. BMC Vet. Res..

[33] Ombarak R.A., Hinenoya A., Elbagory A.-R.M., Yamasaki S. (2018). Prevalence and Molecular Characterization of Antimicrobial Resistance in *Escherichia coli* Isolated from Raw Milk and Raw Milk Cheese in Egypt. J. Food Prot..

[34] Rodrigues L.F.S., Melo R.A., Borges N.M., Aragao A.C.S., Araujo M.O., Dos Santos R.D., Bilac C.A., Gomes K.O., do Prado B.A., Sa Barreto L.C.L. (2025). Antimicrobial Resistance Phenotypes and Genotypes of *Escherichia coli* Isolates from Artisanal Minas Frescal Cheeses from the Federal District, Brazil. Antibiotics.

[35] Tabaran A., Mihaiu M., Tăbăran F., Colobatiu L., Reget O., Borzan M.M., Dan S.D. (2017). First study on characterization of virulence and antibiotic resistance genes in verotoxigenic and enterotoxigenic *E. coli* isolated from raw milk and unpasteurized traditional cheeses in Romania. Folia Microbiol..

[36] Jena J., Sahoo R.K., Debata N.K., Subudhi E. (2017). Prevalence of TEM, SHV, and CTX-M genes of extended-spectrum β-lactamase-producing *Escherichia coli* strains isolated from urinary tract infections in adults. 3 Biotech.

[37] Pandit R., Awal B., Shrestha S.S., Joshi G., Rijal B.P., Parajuli N.P. (2020). Extended-Spectrum β-Lactamase (ESBL) Genotypes among Multidrug-Resistant Uropathogenic *Escherichia coli* Clinical Isolates from a Teaching Hospital of Nepal. Interdiscip. Perspect. Infect. Dis..

[38] Cruz M.C., Hedreyda C.T. (2017). Detection of plasmid-borne β-lactamase genes in extended- spectrum β-lactamase (ESBL) and non-ESBL-producing *Escherichia coli* clinical isolates. Philipp. J. Sci..

[39] Sah R.K., Dahal P., Parajuli R., Giri G.R., Tuladhar E. (2024). Prevalence of *bla*(CTX-M) and *bla*(TEM) Genes in Cefotaxime-Resistant *Escherichia coli* Recovered from Tertiary Care at Central Nepal: A Descriptive Cross-Sectional Study. Can. J. Infect. Dis. Med. Microbiol..

[40] Goleanu Vasiloiu C.D., Vrancianu C.O., Goleanu D.A., Tantu M.M., Csutak O. (2025). Trends in Antibiotic Resistance of *Escherichia coli* Strains Isolated from Clinical Samples *(*2019–2023): A Hospital-Based Retrospective Analysis. Pathogens.

[41] Veenemans J., Overdevest I.T., Snelders E., Willemsen I., Hendriks Y., Adesokan A., Doran G., Bruso S., Rolfe A., Pettersson A. (2014). Next-generation sequencing for typing and detection of resistance genes: Performance of a new commercial method during an outbreak of extended-spectrum-beta-lactamase-producing *Escherichia coli*. J. Clin. Microbiol..

[42] Wang H., Ye C., Jiang H., Song R. (2025). Application of next-generation sequencing in the detection of antimicrobial resistance. Clin. Chim. Acta.

[43] Husz L.H., Tornyos G.Á., Kaszab E., Fehér E., Bittsánszky A., Tóth A.J., Süth M., Jerzsele Á., Kerek Á. (2026). Phenotypic and Genomic Analysis of Antimicrobial Resistance in *Escherichia coli* Isolated from Food-Transport Containers Used in Institutional Catering. Antibiotics.

[44] Ali M., Reshad R., Aunkor M., Biswas G., Mahmud A., Miah M. (2023). Antimicrobial Resistance: Understanding the Mechanism and Strategies for Prevention and Control. J. Adv. Biotechnol. Exp. Ther..

[45] Nunez-Samudio V., Pimentel-Peralta G., De La Cruz A., Landires I. (2024). Multidrug-resistant phenotypes of genetically diverse *Escherichia coli* isolates from healthy domestic cats. Sci. Rep..

[46] Hudzicki J. (2009). Kirby-Bauer-Disk-Diffusion-Susceptibility-Test-Protocol.

[47] Cebeci T. (2022). Prevalence, Characterization and PFGE profiles of multidrug resistance extended spectrum β-lactamase producing *Escherichia coli* strains in animal-derived food products from public markets in Eastern Turkey. J. Hell. Vet. Med. Soc..

[48] Randall L.P., Cooles S.W., Osborn M.K., Piddock L.J., Woodward M.J. (2004). Antibiotic resistance genes, integrons and multiple antibiotic resistance in thirty-five serotypes of Salmonella enterica isolated from humans and animals in the UK. J. Antimicrob. Chemother..

[49] Pishtiwan A.H., Khadija K.M. (2019). Prevalence of *bla*TEM, *bla*SHV, and *bla*CTX-M Genes among ESBL-Producing Klebsiella pneumoniae and *Escherichia coli* Isolated from Thalassemia Patients in Erbil, Iraq. Mediterr. J. Hematol. Infect. Dis..

[50] Ojdana D., Sacha P., Wieczorek P., Czaban S., Michalska A., Jaworowska J., Jurczak A., Poniatowski B., Tryniszewska E. (2014). The Occurrence of *bla*CTX-M, *bla*SHV, and *bla*TEM Genes in Extended-Spectrumβ-Lactamase-Positive Strains of Klebsiella pneumoniae, *Escherichia coli*, and Proteus mirabilisin Poland. Int. J. Antibiot..

